# Global research into the relationship between electronic waste and health over the last 10 years: A scientometric analysis

**DOI:** 10.3389/fpubh.2022.1069172

**Published:** 2023-01-04

**Authors:** Huiting Tian, Lingzhi Chen, Jinyao Wu, Daitian Zheng, Qiuping Yang, Zeqi Ji, Jiehui Cai, Yexi Chen, Zhiyang Li

**Affiliations:** Department of Thyroid, Breast, and Hernia Surgery, General Surgery, The Second Affiliated Hospital of Shantou University Medical College, Shantou, China

**Keywords:** electronic waste (e-waste), health, environment, scientometric, bibliometrix, VOSviewer

## Abstract

**Introduction:**

The aims of this research were to conduct the first holistic and deep scientometric analysis of electronic waste and health and provide with the prediction of research trends and hot topics.

**Method:**

A comprehensive literature search was conducted via the Web of Science Core collection databases on 26 August 2022 to identify all articles related to electronic waste and health. A total of 652 records have been extracted from the Web of Science after applying inclusion and exclusion criteria and were analyzed using bibliometrix software of R-package, VOSviewer, and CiteSpace, visualized by tables and diagrams.

**Result:**

The number of publications and total citations had shown a general growth trend from 2012 to 2021, with an average annual growth rate of 23.74%. Mainland China was the significant nation with the greatest number of publications, citations, and international links. The journal publishing the most was “Science of the Total Environment” (*n* = 56). Huo X and Hu XJ were the top two author contributing to this field with the highest *h*-index (23). Over time, the focus in this field shifted to exposure to heavy metal, polychlorinated biphenyls, polybrominated biphenyl ethers, and poly- and perfluorinated alkyl substances from electronic waste, and managements, such as hydrometallurgy.

**Discussion:**

By this scientometric analysis, we found that the most active country, journal, organization and author contributing to this filed, as well as high impact documents and references and research hotspots. Also, we found that the hotspots might be exposure to toxic substances from electronic waste procession, its impact on human health and relevant managements. And evironmentally friendly materials to replace heavy metal mate rials, and environmentally friendly and effective recycling methods of electronic waste need to be further studied.

## Introduction

Population growth, technological advancement, and economic expansion accelerated the rise in demand for electronic goods and shortened their replacement cycles. Due to the increase in the disposal of end-of-life (EoL) electrical and electronic goods, electrical and electronic waste (e-waste) contribute as one of the major pollution-causing products. Without proper management, the presence of heavy metals such as Hg, Cd, Pb, Co, Ni, Ti, Ag, Hg, Cd, As, and brominated flame retardants (BFRs), as well as other potentially dangerous compounds in e-waste, poses a threat to both the environment and human health (1–13).

A study by Gwenzi et al. revealed that high-tech rare earth elements (REEs) of anthropogenic origin are found in the environment, including aquatic systems. The probability of developing nephrogenic systemic fibrosis, severe kidney damage from Gd-based contrast media, dysfunctional neurological disorder, fibrotic tissue injury, peroxidation, lung damage, cytotoxicity, anti-testicular effects, and infertility may all increase with human exposure to REEs (14). The studies by Grant et al. (15) and Issah et al. (16) showed that exposure to electronic waste may lead to changes in thyroid function, cell expression and function, temperament and behavior, cause adverse neonatal outcomes, decrease lung function, and even damage DNA. Some studies found that excessive exposure to lead is associated with multisystem and long-term effects in neonates and children, including neurological, cardiovascular, adaptive immune, and blood systems, as well as chromosomal and DNA damage (17–19).

In summary, e-waste has become a problem influencing human health that needs urgent attention. Several studies on electric waste and health are available. A scientometric analysis is particularly suitable for measuring a field as a whole and providing an overview of the state, scope, and impact of the field and the major contributors involved. However, there have been no studies that conducted research using the scientometric analysis in this field. This study is the first to study the research trend of e-waste, toxic products from recycling and processing e-waste, and health and provide some valuable insights to future researchers.

## Materials and methods

### Data collection

The Web of Science Core Collection database of Clarivate Analysis was used to search for all pieces of literature on the connection between electronic waste and health. Search tactics included the use of the medical subject headings (mesh) as well as the entry phrases “electronic waste” and “health.” We first used the online “analyze results” feature of the Web of Science (WoS) to learn more about the publication years, types of documents, authors, affiliations, sources, countries, languages, and open-access texts.

The retrieval formula was as follows: #1, TS = (“Electronic Waste^*^”) OR TS = (“Waste^*^, Electronic”) #2, TS = (“Health^*^”) or TS = (“Normal^*^”); #3, #1, and #2. Apart from that, the timespan of these publications was filtered from 2012 to 2022. On 25 August 2022, the research was completed, yielding a total of 669 publications. Then, we restricted the language to English and the document types to articles or reviews, yielding a total of 652 publications, including 519 articles and 133 reviews (Table 1). Twenty-three languages other than English and non-publications were excluded. Two duplicate articles were detected using the Zotero software and eliminated. Finally, 519 articles from 652 periodicals, representing 79.60% of the total, and 133 review articles (20.40%) were included in the scientometric study.

**Table 1:** Main information of screened records.

**Description**	**Results**
**Main information about data**	
Timespan	2012:2022
Sources (Journals, Books, etc.)	230
Documents	652
Annual growth rate (2012-2021) %	23.74
Document average age	3.57
Average citations per document	26.87
References	29,891
**Document contents**	
Keywords plus (ID)	1,741
Author's keywords (DE)	1,739
**Authors**	
Authors	2,474
Authors of single-authored documents	29
**Authors collaboration**	
Single-authored documents	32
Co-authors per documents	5.48
International Co-authorships %	39.88
**Document types**	
Article	504
Article; data paper	2
Article; early access	7
Article; proceedings paper	6
Review	127
Review; book chapter	1
Review; early access	5

### Inclusion criteria

**Figure 1:**
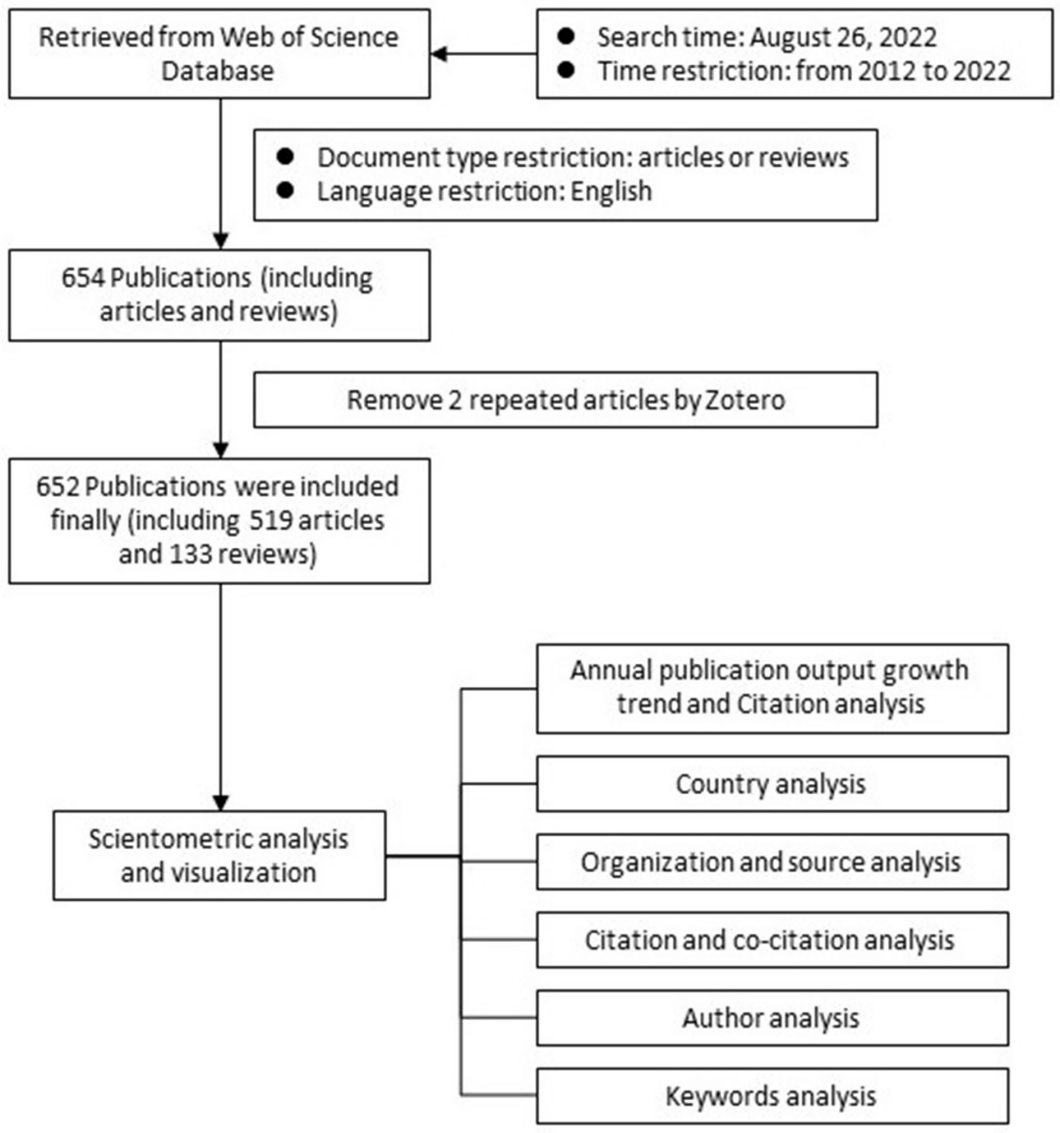
Data collection and the analysis flowchart.

The inclusion criteria were as follows: (1) articles searched from WoS; (2) article types were “article” or “review”; (3) articles published from 2012 to 2022; (4) the language of the articles was English. Figure 1 displays the procedures used to gather the documents and analyze the data. This study needed no ethics committee approval.

### Exclusion criteria

The exclusion criteria were as follows: (1) non-English articles, non-original articles, and non-reviews; (2) retracted articles; and (3) duplicate articles.

### Visualization and scientometrics analysis

The information from these documents was loaded into the Biblioshiny website, CiteSpace, and VOSviewer.

An R-tool of R-studio (version 4.2.1), Bibliometrix, was used for a comprehensive science mapping analysis to provide the description, evaluation, and monitoring of published research (20). It was used to convert and analyze publications, citations, and sources of information. The raw files of the retrieved data were imported into the Bibliometrix to obtain the main information (Table 1): annual scientific production, the most productive countries' contribution, the collaboration between the most productive countries, sources (most relevant sources, impact of sources, growth dynamics of sources), authors (production of authors over time, impact of authors ranked by the *h*-index, *g*-index, *m*-index, and the total number of citations), most cited documents and references, and keywords. The co-occurrence network of trend topics across years was used as a metric to help detect the focus of hotspot research.

CiteSpace provides a quantitative and qualitative evaluation of the literature in the field based on a brief analysis of the state of research, research priorities, and evolution of the field (21). Through a CiteSpace visual analysis, the top 20 references with the strongest citation explosion from around the world are compared and analyzed to explore the current status of research and future trends in the field on a global scale.

VOSviewer is a tool that creates maps using network data to build networks of scientific sources, scientists, research organizations, countries, and keywords. It supports three map visualizations: network visualization, overlay visualization, and density visualization (22). Networks were built using VOSviewer (version 1.6.18): country co-authorship, organization co-authorship, references co-citation analysis, document citation analysis, and keyword co-occurrence analysis. The overlay map showed the co-authorship of countries, and the co-citation of references was also shown by the density map. Further, the keywords occurring more than five times were shown in the three visualizations of the co-occurrence analysis to identify important terms (23).

The *h*-index is an author's or journal's number of published articles, which is widely used to quantify and standardize researchers' scientific impact. Each of these published articles has been cited in another paper at least one time. The *M*-index is defined as *h*/*n*, where *h* is the *h*-index and *n* is the number of years since the year of the first paper of the scientist or journal published, which will remove the effects of different academic career lengths. Using *g* as the serial number, the articles published by authors were arranged in descending order of the number of citations. The sum of the top *g* articles' citations is at least a *g*^2^ citation. A high *g*-index indicates a high citation rate (24–26).

## Results

### Annual publication output growth trend and citation analysis

**Figure 2:**
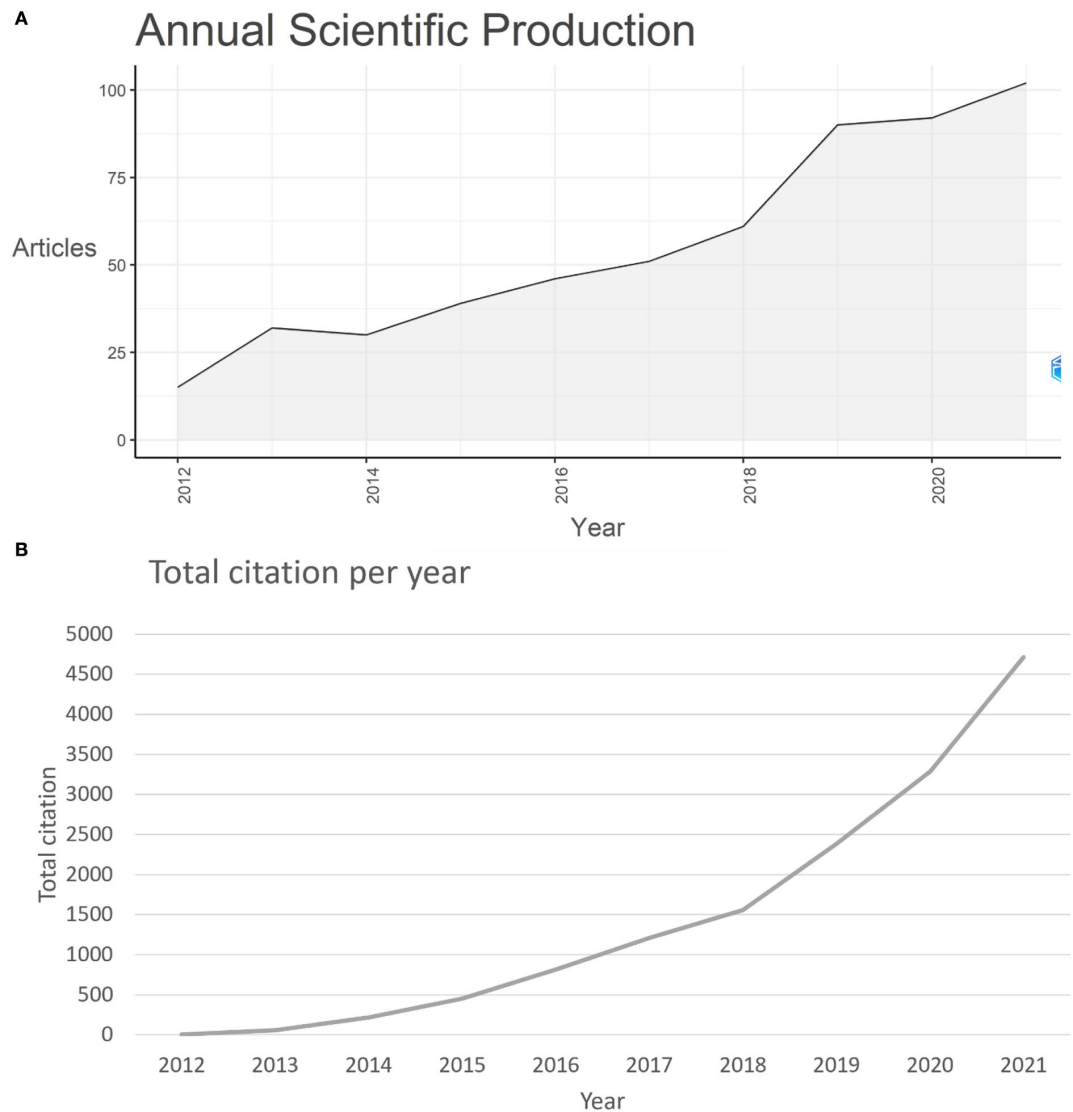
**(A)** Annual scientific production during the last 10 years in this field; **(B)** Total article citations per year during the last 10 years in this field.

Over the last 10 years, 2012–2021, the total number of documents retrieved from WoS related to this topic was 558, as shown in Figure 2A, and the annual growth rate was 23.74%. Further, 2021 was the year with the highest contribution to publication output (102, 18.27%). There was an increasing trend from 15 publications in 2012 (2.68%) to 102 publications in 2021 (18.27%), showing a general growth trend. From 2016 to 2019, the growth rate increased from 10 to 19%. The highest growth rate was observed in 2012, from 15 to 32 articles (113.33%), and the growth rate of articles decreased in 2022 (2.22%) and increased in 2021 (10.87%). As shown in Figure 2B, the total article citations grew in a general growth trend from 4 citations in 2012 to 4,715 in 2021.

### Countries analysis

**Figure 3:**
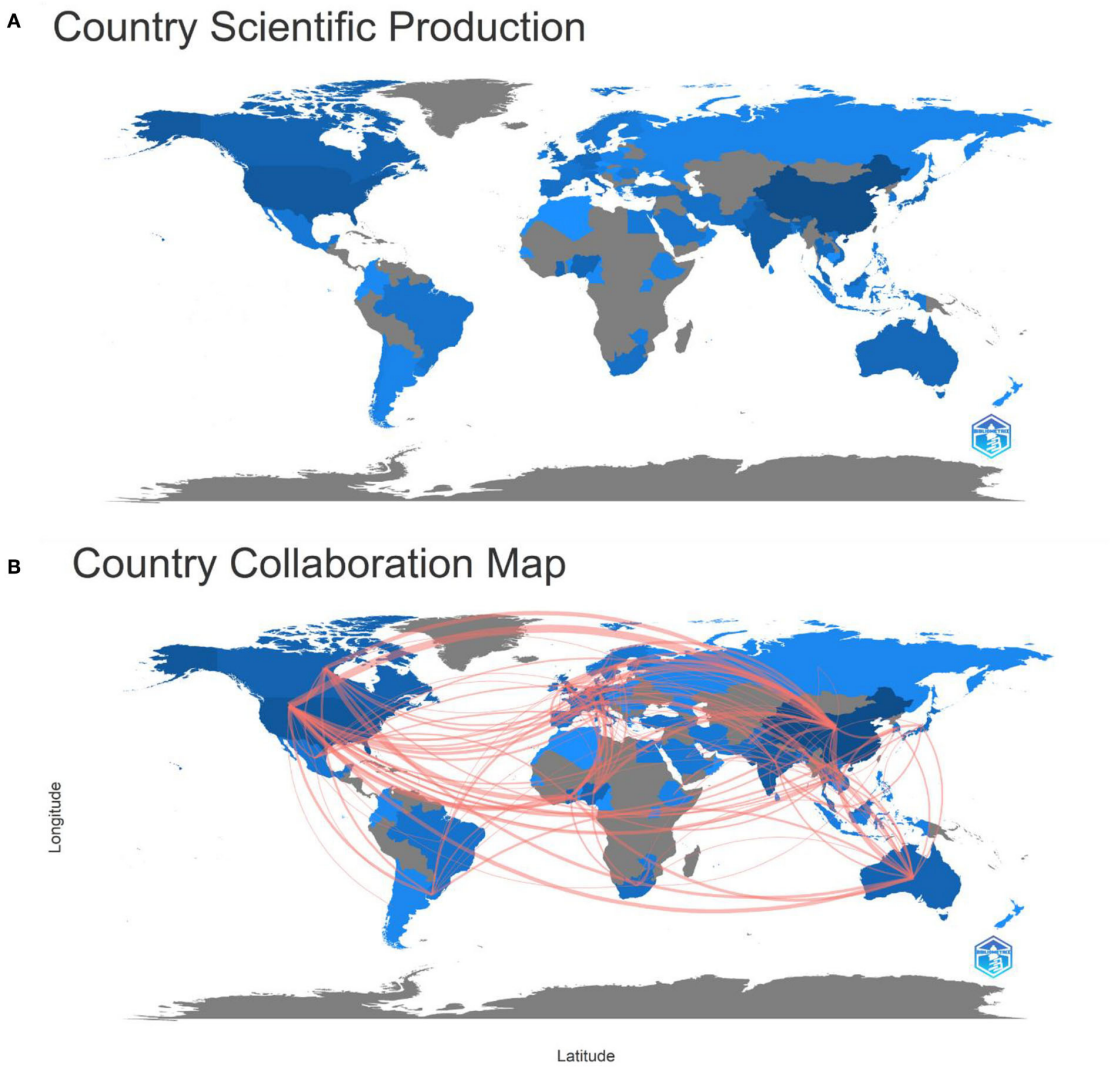
**(A)** Country or region scientific production world map; **(B)** country or region collaboration world map (blue color: country or region with publications; gray color: country or region without publications; the intensity: the publications' number).

In the 652 publications analyzed, the authors lived in 79 countries or territories, as displayed in Figure 3A. A country or territory colored blue on a map of the globe indicates that authors from these countries have contributed to the literature; the darker the blue hue, the greater the number of articles produced from that country or region. It was evident that China (excluding Hong Kong, Macau, and Taiwan, henceforth), the United States, and India are all darker than any other nation or region, with 44.17, 17.79, and 11.04% of all publications, respectively. There are more countries outside these three where publications account for over 2% of the total number of countries, such as Canada, Ghana, Australia, Nigeria, the Netherlands, England, Malaysia, Germany, Thailand, South Africa, Japan, Vietnam, Pakistan, and Belgium. The main participants include seven Asian countries, five European countries, two North American countries, and three African countries.

Figure 3B shows the graph of cooperation between countries or regions. If there are any partnerships between two nations or regions, they are linked by red lines whose thickness is positively correlated with the number of partnerships. Figure 3B shows several red lines connecting mainland China, the USA, and other nations or regions, suggesting that these two nations were the publication's focal points. The number of partnerships between the USA and mainland China results in 41 co-publications, making up the greatest thickness.

Table 2 shows the top 10 countries contributing to this field of study. China was the top contributor in this field, with the highest total of citations, 9,736, which is far more than any other country. The United States followed with 3,353 citations, followed sequentially by Australia (*n* = 2008), India (*n* = 1833), Canada (*n* = 1098), and the Netherlands (*n* = 1041).

**Table 2:** Top 10 cited countries contributing to this research area.

**Country**	**Documents**	**Total citations**	**Total link strength**
Peoples R China	288	9,736	166
USA	116	3,353	169
India	72	1,833	54
Canada	43	1,098	89
Ghana	39	857	85
Australia	37	2,008	91
Nigeria	31	496	46
Netherlands	27	1,041	67
England	25	467	47
Malaysia	23	565	29

**Figure 4:**
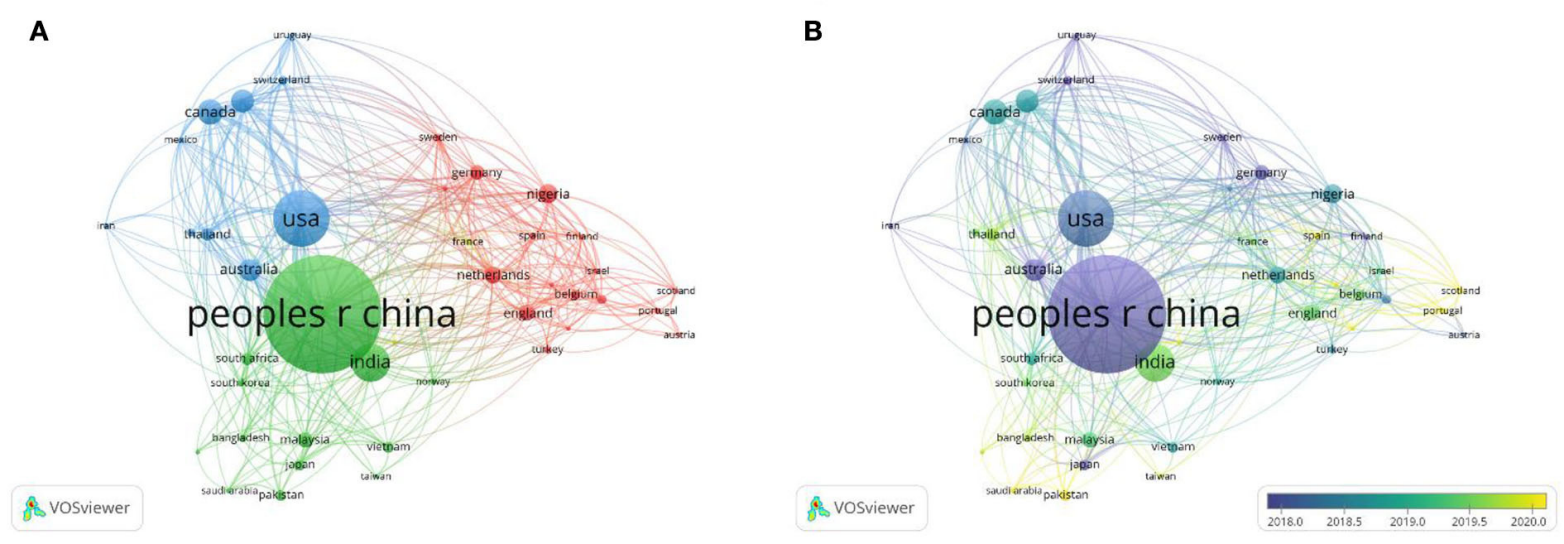
**(A)** Co-authorship network map between countries with over five publications; **(B)** Co-authorship overlay map between countries with over five publications (the blue color stands for earlier years; the yellow color stands for more recent years).

A total of 42 nations with much more than five publications in this field of study were examined in the co-authorship analysis. The USA (total link strength = 169), China (166), Australia (91), Canada (89), Ghana (85), and the Netherlands (67) were the top five nations in terms of total link strength (Figure 4A). The diameter of the circles depicts the overall strength of ties between the various nations, and the distance between the circles denotes the strength of the linkages based on how frequently they occur. With time, certain developing nations started to show startling data in this area, including Ghana (total link strength = 85), India (54), Nigeria (46), and Malaysia (29; Figure 4B).

### Organizations and source analysis

**Figure 5:**
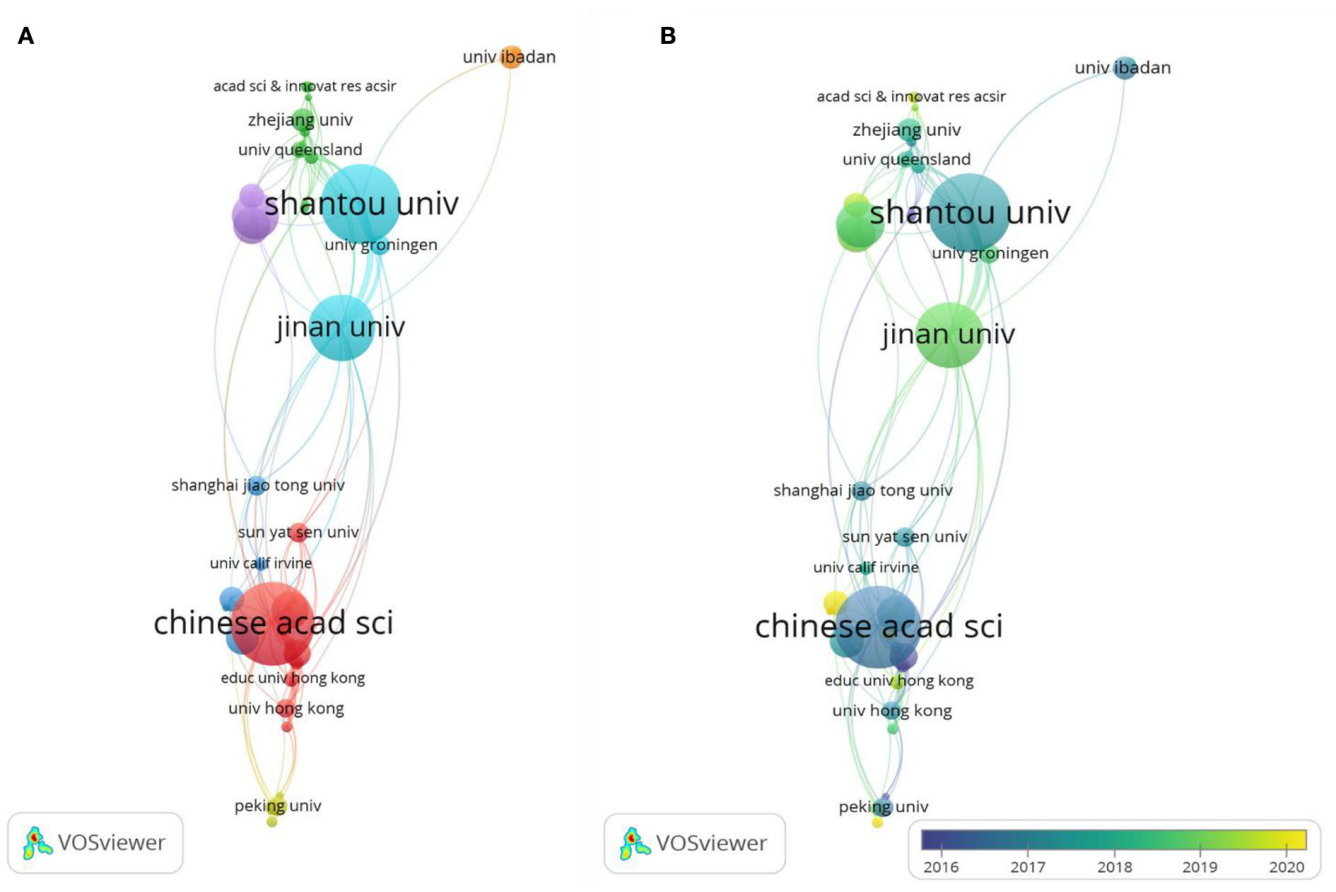
**(A)** Co-authorship network map for organizations with over five publications; **(B)** Co-authorship overlay map for organizations with over five publications.

A total of 967 organizations were involved in this field. The Chinese Academy of Sciences contributed the most publications (*n* = 49, 7.52%), followed by Shantou University (*n* =47, 7.2%), Jinan University (*n* = 39, 5.98%), the University of Ghana (*n* = 28, 4.29%), and the University of Michigan (*n* = 22, 3.37%; Table 3, Figure 5A). Then, we analyzed the organization's co-authorship of over five papers and obtained the results for 42 institutions. The top five institutions in terms of the strength of their total links were Jinan University (59), Shantou University (58), the Chinese Academy of Sciences (56), the University of Ghana (30), and the University of Chinese Academy of Sciences (20; Table 3, Figure 5A). Over time, the average years of contributions in this field by these organizations are closer to the recent, such as Nanjing Medical University, Guangdong University of Technology, South China Normal University, Synergy Innovation Institution GDUT, Kwame Nkrumah University of Science and Technology, the Academy of Scientific and Innovative Research, and so on (Figure 5B).

**Table 3:** Most productive organizations contributing to this area.

**Organizations**	**Documents**	**Citations**	**Total link strength**
Chinese Academy of Sciences	49	1,959	56
Shantou University	47	1891	58
Jinan University	39	927	59
University of Ghana	28	490	30
University Michigan	22	374	22
Tsinghua University	22	1,592	15
University of Chinese Academy Science	20	643	25
Hong Kong Baptist University	16	757	24
Mcgill University	15	190	22
Guangdong University Technol	15	188	12

These 652 papers were published in 230 journals. The top 10 journals in this discipline regarding productivity are shown in Supplementary Table 1. A total of 280 papers were published in these top 10 journals (42.94% of all). “Science of the Total Environment” published papers with the maximum number (*n* = 56). The second-ranked journal was “Environmental Science and Pollution Research” (*n* = 44). “Environment International” ranked third (*n* = 32), followed by “Chemosphere” (*n* = 27) and “Environmental Pollution” (*n* = 26). These journals focused on environmental science. “Science of the Total Environment” had the highest number of citations (1,820), followed by “Waste Management” (1,611 citations), “Environment International” (1,220 citations), “Journal of Cleaner Production” (1,089 citations), and “Environmental Pollution” (1,080 citations). The significance of these journals was discussed in terms of source impact using the *h*-index (25, 26). The *h*-index for “Science of the Total Environment” was the largest at 24, followed by “Environment International” (*h*-index = 22) and “Environmental Science and Pollution Research” (*h*-index = 19; Table 4). “Chemosphere,” “Environment International,” “Environmental Pollution,” “Environmental Science and Pollution Research,” and “Science of the Total Environment” have been active in this field for many years. “Environment International” rapidly developed in 2015 and overtook “Environment Pollution” and “Chemosphere” in 2019 (Supplementary Figure 1).

**Table 4:** Impact of sources on the top 10 journals in this field.

**Journal**	***H*-index**	***G*-index**	***M*-index**	**Total citations**	**Number of publications**	**Publication year_start**
Science of the Total Environment	24	42	2.182	1,820	53	2012
Environment International	22	31	2	1,220	31	2012
Environmental Science and Pollution Research	19	28	1.9	856	39	2013
Environmental Pollution	18	24	2	1,080	24	2014
Chemosphere	14	25	1.4	845	25	2013
Journal of Cleaner Production	14	17	1.4	1,089	17	2013
Waste Management	13	20	1.3	1,611	20	2013
Resources Conservation and Recycling	12	15	1.2	733	15	2013
International Journal of Environmental Research and Public Health	10	16	1.429	282	20	2016
Journal of Hazardous Materials	10	16	0.909	435	16	2012

### Citation and co-citation analysis

**Figure 6:**
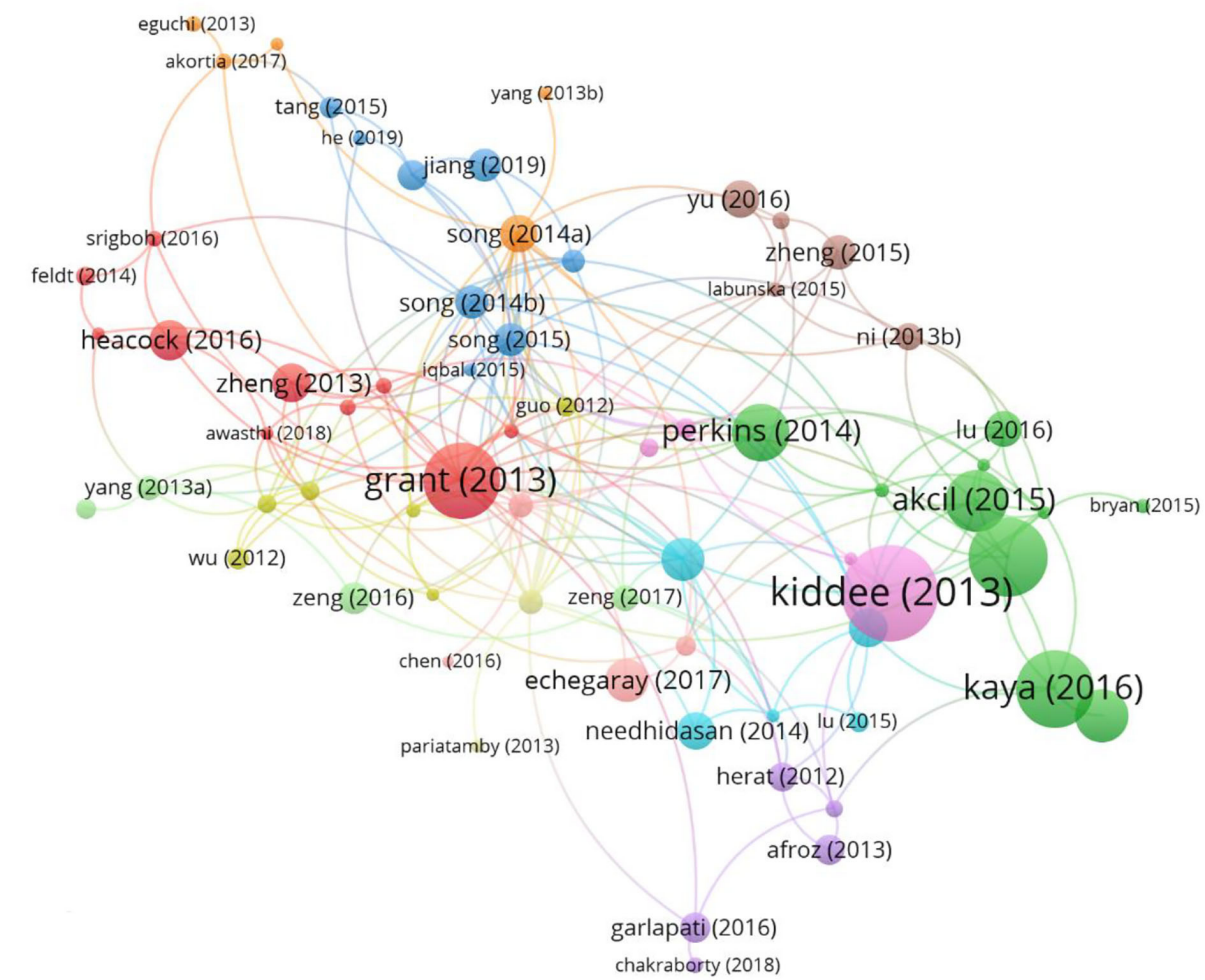
Citation analysis of documents network map with over 50 citations.

The citation analysis showed that 89 documents were cited more than 50 times (Figure 6). The top ten most-cited documents are listed in Table 5. Among them, “Electronic waste management approaches: an overview” (7) had 382 citations, followed by “A wearable transient pressure sensor made with MXene nanosheets for sensitive broad-range human-machine interfacing” (27) with 327 citations. The third-most-cited article was “Waste Printed Circuit Boards Recycling: An Extensive Assessment of Current Status” (28), with 316 citations.

**Table 5:** Top 10 global cited documents.

**Paper**	**DOI**	**Publication year**	**Total citations**
Electronic waste management approaches: an overview	doi: 10.1016/j.wasman.2013.01.006	2013	382
A wearable transient pressure sensor made with MXene nanosheets for sensitive broad-range human-machine interfacing	doi: 10.1021/acs.nanolett.8b04514	2019	327
Waste printed circuit boards recycling: an extensive assessment of current status	doi: 10.1016/j.jclepro.2015.02.024	2015	316
Recovery of metals and nonmetals from electronic waste by physical and chemical recycling processes	doi: 10.1016/j.wasman.2016.08.004	2016	309
Health consequences of exposure to e-waste: a systematic review	doi: 10.1016/S2214-109X(13)70101-3	2013	306
Precious metal recovery from waste printed circuit boards using cyanide and non-cyanide lixiviants – a review	doi: 10.1016/j.wasman.2015.01.017	2015	247
Potential environmental and human health impacts of rechargeable lithium batteries in electronic waste	doi: 10.1021/es400614y	2013	246
E-waste: a global hazard	doi: 10.1016/j.aogh.2014.10.001	2014	232
Sources, behavior, and environmental and human health risks of high-technology rare earth elements as emerging contaminants	doi: 10.1016/j.scitotenv.2018.04.235	2018	210
Nickel recovery/removal from industrial wastes: a review	doi: 10.1016/j.resconrec.2013.01.019	2013	185

**Figure 7:**
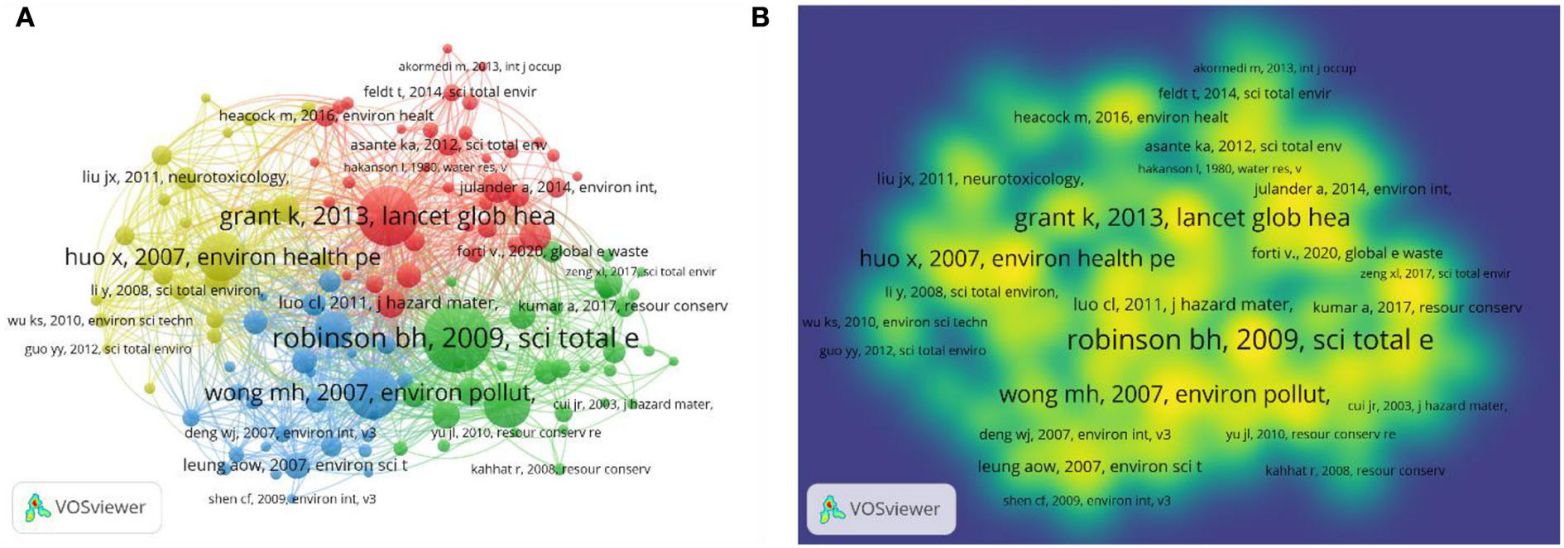
**(A)** Co-citation network map between references with over 20 citations; **(B)** Co-citation density map between references with over 20 citations.

A co-citation network map, conducted with VOSviewer, of these publications' references is shown in Figure 7. Using 20 as the minimal citation count for the references, 123 references met the requirement, and two unidentified references were excluded. Those two unidentified references had a total of 114 citations, which may not necessarily be from the same two articles. The node grows in size as more references are cited (Figure 7A). A deeper yellow tint indicated more citation (Figure 7B). Figure 7A presents the five groups of cited sources. The top group, highlighted in red and with 38 entries, represents the most desirable study areas. With 141 citations, Robinson's (29) paper was the most cited, as illustrated in Figure 7. This paper mainly indicated two points. First, there were valuable metals and potential environmental pollution, such as Pb, Hg, Sb, and polychlorinated biphenyls (PCBs). Second, using the unsuitable handling method of processing e-waste could generate toxic substances, e.g., dioxins, impacting human health (29). The top five most cited references were Robinson (29) (141 citations), Grant et al. (15) (122 citations), Wong et al. (30) (106 citations), Huo et al. (31) (99 citations), and Widmer et al. (32) (95 citations). We identified the top 20 references with the most powerful citation explosions *via* CiteSpace (Supplementary Figure 2). Notably, 65% of them (12/20) showed a citation burst in the period 2014–2016, followed by those in the period 2017–2019 (7/20, 40%). In addition, two references, Balde (33) and Srigboh (34), were cited from 2019 in rapid succession.

### Author analysis

**Figure 8:**
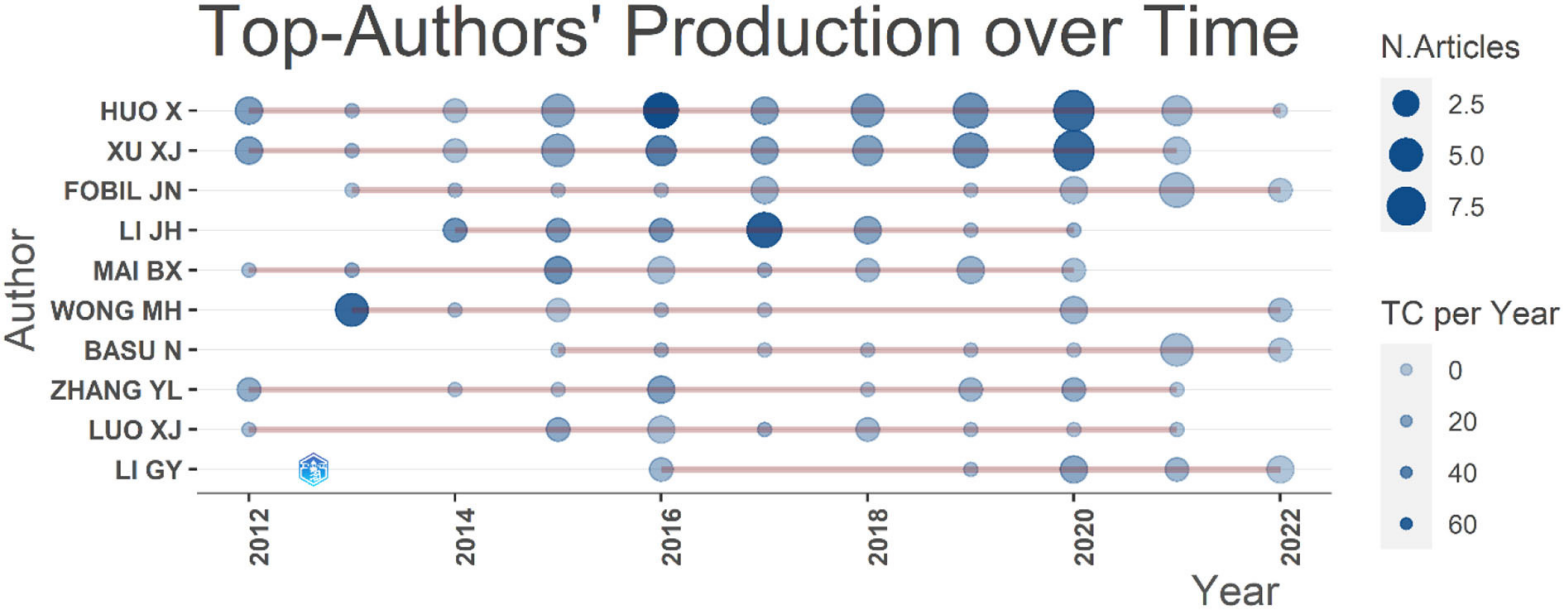
Top authors' production over time.

A total of 2,474 writers−32 writers of single-authored publications and 2,442 writers of multiple-authored publications—produced these 652 documents. There were 32 single-authored documents. The mean number of co-authors per paper is 5.48. Huo X was the most productive author with 45 publications, accounting for 6.90% of the total, followed by Xu XJ with 40 publications (6.13%), and then Fobil JN (*n* = 20, 3.07%; Supplementary Figure 3A). Meanwhile, Huo X and Xu XJ were the authors with the highest *h*-index (*h* index = 23; Table 6). Authors with higher productivity and a higher *h*-index held more prominence in the area. Sixteen authors had written more than 10 pieces, while three authors had written more than 20. In Figure 8, the number of articles was represented by the size of the dots, and the overall number of citations each year was represented by the gradation of the color of the dots. Huo X and Xu XJ maintained a steady tendency. Fobil JN, Basu N, and An TC were more active in publishing in the last 3 years.

**Table 6:** Top 10 contributing authors in this area.

**Author**	***h*-index**	**No of articles**	***g*-index**	***m*-index**	**Total citation**	**Publication year_start**
Huo X	23	45	40	2.091	1,652	2012
Xu XJ	23	40	37	2.091	1,418	2012
Li JH	17	17	17	1.889	1,229	2014
Zhang YL	12	13	13	1.091	489	2012
Mai BX	11	16	16	1	716	2012
Wong MH	10	15	15	1	717	2013
Fobil JN	9	20	17	–	315	–
Liu Y	8	10	9	1.333	226	2017
Awasthi AK	7	8	7	1	457	2016
Chen AM	7	7	7	1	399	2016

According to the *h*-index (Table 6), Huo X and Xu XJ ranked the first (*h*-index = 23), Li JH ranked second (17), Zhang YL (12) ranked third, Mai BX (11) ranked fourth, followed by Wong MH (10). The top five authors are listed in Supplementary Figure 3B in order of the number of citations their articles received. Huo X ranked first with 1,652 citations, followed by Xu XJ (1418 citations), Li JH (1,229 citations), Sly PD (856 citations), and Wong MH (717 citations). Among the most productive authors, six had an *h*-index above 10. The *m*-index, which takes various seniorities of scientists into consideration, corrects the *h*-index through time and aids in the identification of successful researchers (25, 26). Huo X and Xu XJ had the highest *m*-index at 2.091, followed by Arya S, Bilal M, and Liu H (*m*-index = 2.000), Li, JH (1.889), Ma ST and Yu YX (1.500), and Liu Y (1.333; Supplementary Figure 3C). We examined 56 researchers who had published over five articles. A higher *g*-index indicates a higher citation rate. Huo X (*g*-index = 40) had the highest *g*-index, followed by Xu XJ (37), Li JH, and Fobil JN (17), as shown in Supplementary Figure 3D. Huo X (total link strength = 112), Xu XJ (99), and Zhang YL (43) were the top three researchers with the greatest total link strength.

### Keywords analysis

**Figure 9:**
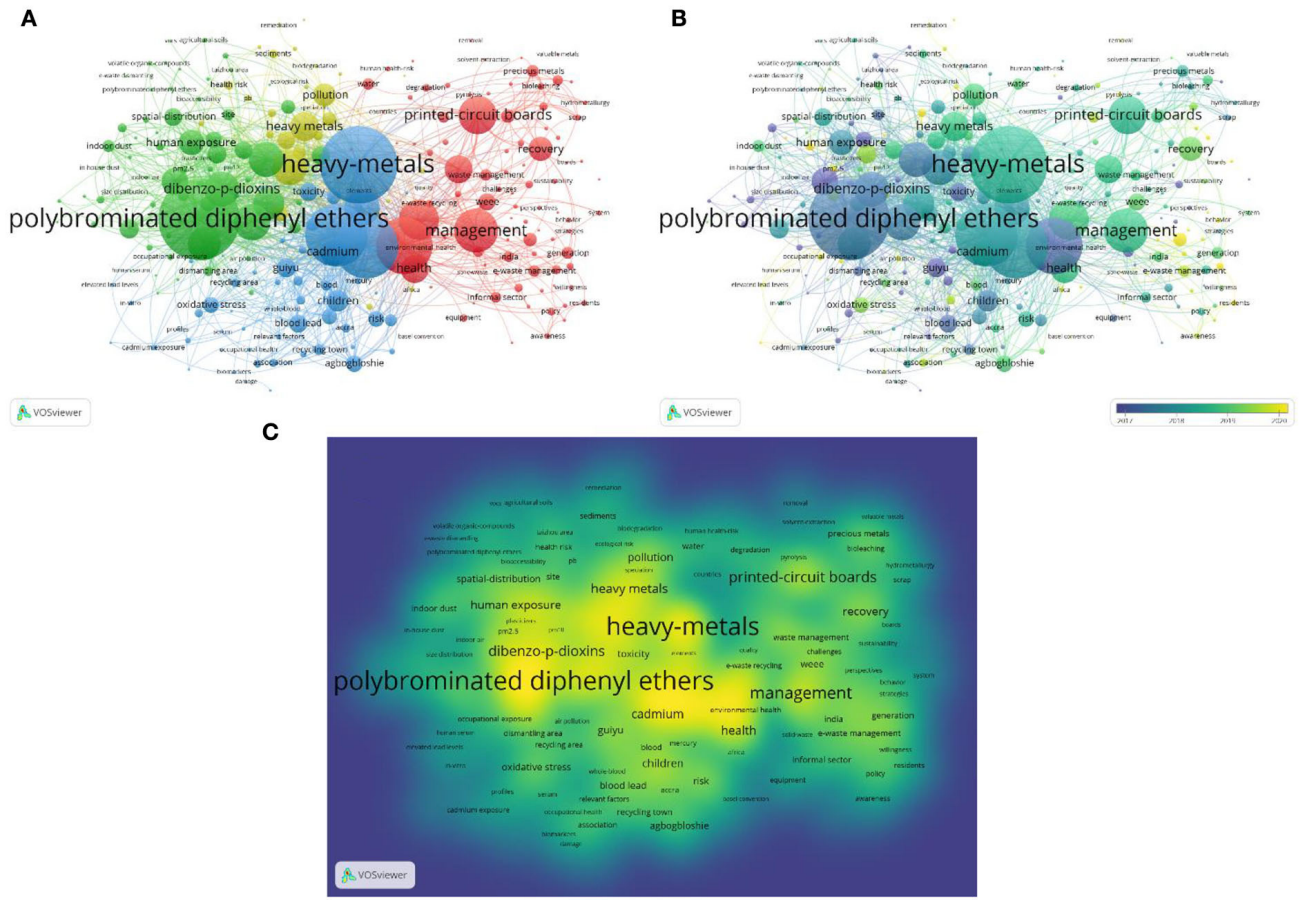
**(A)** Keywords network map with over five occurrences; **(B)** Keywords overlay map with over five occurrences; **(C)** Keywords density map with over five occurrences.

**Figure 10:**
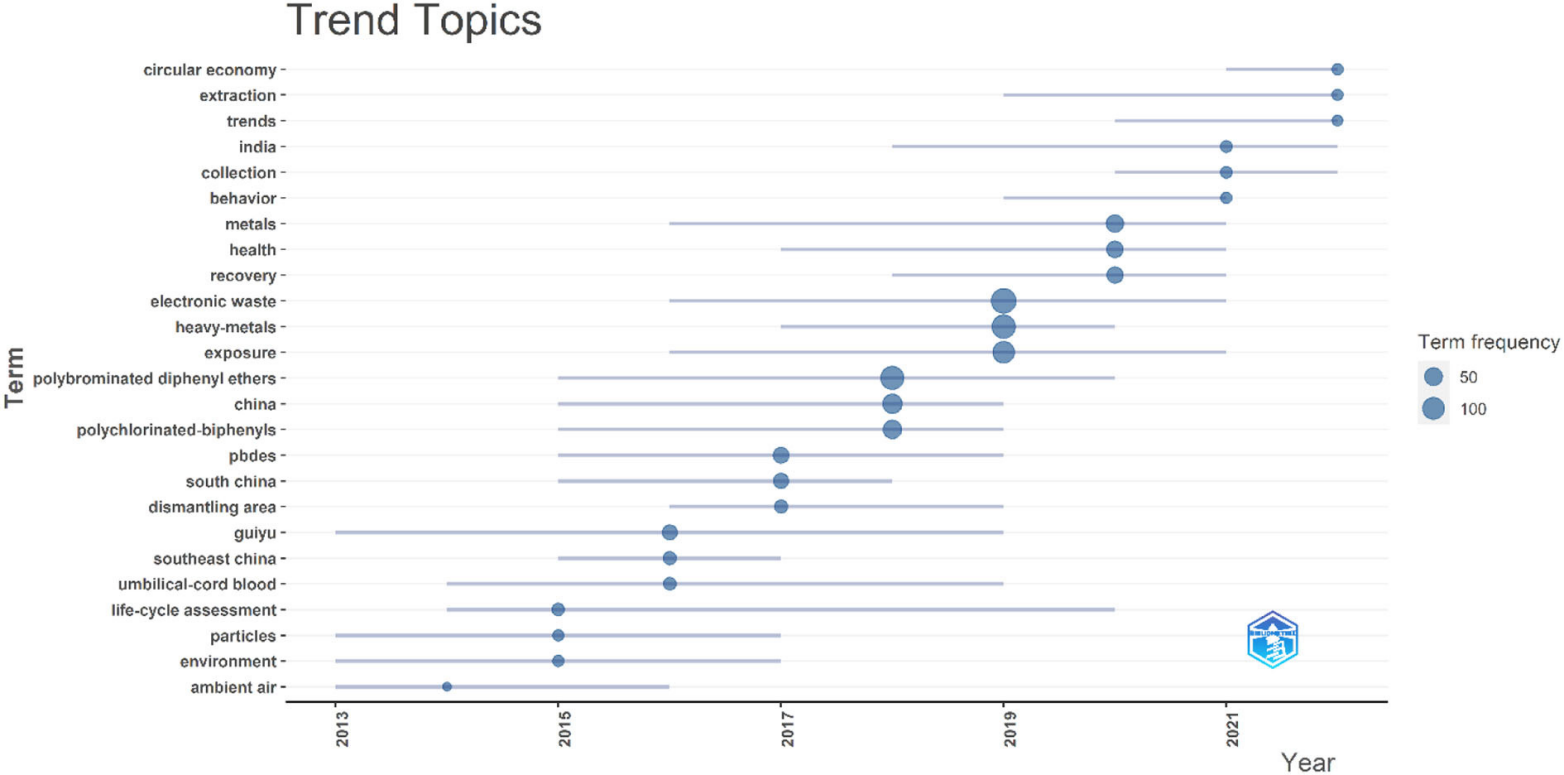
Map showing the trending topics across years.

VOSviewer and Bibliometrix examined 266 terms with over five appearances, and we excluded the search terms “e-waste” and “electronic waste.” We divided the entire co-occurrence network into various groups using VOSviewer's grouping feature. Stronger, relevant keywords were more likely to be clustered with the same hues. These chosen keywords were categorized into four groups, as seen in the network visualization graph (Figure 9A). In addition, the hue of the superimposed visual map shows the average publication year of these keywords (Figure 9B). If the keyword appeared averagely later, the hue is closer to yellow. To find the topic of the current research, the keywords with the highest frequencies in the most publications were displayed on the graph of keyword density (Figure 9C). When the keywords occur in more publications, the hue is closer to yellow. “Polybrominated diphenyl ethers” (*n* = 129), “heavy metals” (*n* = 123), “exposure” (*n* = 101), “China” (*n* = 90), “management” (*n* = 70), and so on were the most used keywords. Over time, the focus of the research field shifted from the relationship between ambient air, chemicals, particles, and lifecycle assessment to the health and circular economy implications of heavy metal exposure and accumulation due to e-waste (Figure 10).

## Discussion

Using Bibliometrix, VOSviewer, and CiteSpace, this paper presented a scientometric analysis of the production of e-waste and health-related publications from 2012 to 2022. At the time of the search, there were 652 pieces of literature on this subject. The number of related publications increased between 2012 and 2021, and the annual growth rate was 23.74%, with the largest growth rate occurring in 2012 (113.33%). There was a general growth trend in publications and total citations, indicating an overall increase in interest in this research area. The growth rate of articles decreased in 2020 (2.22%). Although there was a slight decrease in 2020, the growth rate rose again in 2021 (10.87%). Up until our search, the number of publications in 2022 was 94. Considering the number of publications and total citations in 2021 (102, 4,715) and the general growth trend, we presumed that the number of productions and total citations would continue to rise in 2022.

China, the United States, and India were the largest contributors in this area and were the epicenter of this research. China was in first place, far ahead of other countries, with 288 articles and a total citation of 9,736, while the United States and India had 116 and 72 articles, with total citations of 3,353 and 1,833, respectively, which shows that, although China is a developing country, it pursues sustainable development and also pays attention to its people's health and ecological balance. The country collaboration map shows that the cooperation among countries was also close, forming a research network centered on China. The top three organizations contributing to this field were the Chinese Academy of Sciences, Shantou University, and Tsinghua University. The total number of citations was more than 1,000, indicating that these three organizations had leading roles in this field and strong connections with other organizations, as measured by their link strength of 376. The top three high-impact journals in this field were “Science of the Total Environment,” “Environment International,” and “Environmental Pollution,” which had a total citation count of over 1,000 and an *h*-index of over 18, indicating that the articles published in these three journals are more influential in this field, worthy of attention, and suitable for reference.

In addition, 50% of the top 10 most cited papers worldwide were published between 2015 and 2019. These ten papers have one thing in common: they all assert that e-waste contains a variety of toxic substances that are harmful to human health, investigate the nature of these substances, and examine potential solutions from multiple perspectives, demonstrating a fundamental understanding of this topic. Toxic substances mainly include dioxin-like compounds (DLCs), polybrominated biphenyl ethers (PBDEs), polychlorinated biphenyls (PCBs), PM2.5, lead (Pb), cobalt (Co), copper (Cu), nickel (Ni), thallium (Ti), and silver (Ag), hydrargyrum (Hg), cadmium (Cd), mercury (Hg), and arsenic (As), high-technology rare earth elements (REEs) of anthropogenic origin, the gases produced (dioxins, furans, polybrominated organic pollutants, and polycyclic aromatic hydrocarbons) by thermal treatments, and brominated flame retardants (BFRs), among others (1, 2, 4–6, 10, 14, 35–39). The study by Tansel et al. indicates that poly- and perfluorinated alkyl substances (PFAS) in e-waste may be a substance that affects human health; however, further study is needed to confirm it. There is a need for better surveillance of e-waste disposal sites, and detailed epidemiological studies of high-risk populations can be undertaken to assess the potential health risks posed by exposure to PFAS at these sites (40).

The following steps can be taken to avoid electronic waste that can be harmful to human health: (1) Prevent the generation of electronic pollutants at the source, identify what hazardous substances are in e-waste sources, their environmental behavior, and their public and ecological health risks, and identify environmentally friendly materials to replace those materials producing hazardous substances; (2) prevent electronic products from turning into electronic pollution by following an intermediate steps: enhance the recycling rate and its management mechanism, improve the life of electronic products, raise people's awareness of sustainability through education, and reduce the generation of electronic pollution; (3) develop a series of pollution treatment mechanisms after the generation of e-waste, such as effective e-waste disposal methods, ecologically responsible management, and routine monitoringv (13, 41–43). Several methods have been built to manage e-waste, including life cycle assessment (LCA), material flow analysis (MFA), multi-criteria analysis (MCA), and extended producer responsibility (EPR). The keys to success in e-waste management are the development of ecologically designed equipment, proper collection of e-waste, recycling of materials by safe methods, disposal of e-waste with appropriate technologies, prohibition of transfer of used and end-of-life electronic equipment to developing countries, and raising awareness of the impacts of e-waste (7, 43–46). The study by Kang et al. used standardized leaching procedures, life cycle impact assessment (LCIA), and hazard evaluation systems to analyze the potential toxicity, potential resource depletion, and hazardous waste categories of electronic products to reduce potential hazards to human health (5). The studies by Kaya et al. and Perkins et al. mentioned that e-waste recycling would become an important industry soon and become an economically and environmentally significant industry. Their aims were to turn today's waste into tomorrow's conflict-free, sustainable polymetallic secondary resource, which requires e-waste to be handled in ecologically responsible, safe, and standardized ways with good efficiency and low carbon emission (6, 47). In addition to the aforementioned entry points, there were others, including the need to integrate e-waste into urban planning efforts through phytoremediation (48, 49).

There are relatively mature physical separation processes, such as gravity, electrostatics, magnetic separators, flotation, and so on, to physically separate and recover metals from e-waste through pyrometallurgy, hydrometallurgy, or biohydrometallurgy. Cyanide or non-cyanide leaching methods of hydrometallurgy are effective methods for recovering precious metals efficiently using non-metallic materials (1, 6, 28, 50). In addition, plasma technology and pyrolysis treatment were applied to handling e-waste (51–53). According to the search, no environmentally friendly electronic materials have yet been found, which can be used as a starting point for further research to find substances that can be recycled in an environmentally friendly manner. Then, we believe that health education and policies are still lacking. We can start by increasing the effectiveness of education and expanding the popularity of environmental protection policies, allocating more government funds for the management and treatment of electronic pollution, centralizing end-of-life processing, improving the electronic product recycling rate, and reducing the generation of e-waste (54, 55).

In the author analysis, Huo X and Xu XJ had the highest number of publications (45, 40) and *h*-index (23, 23). The consistency and magnitude of their contributions over time indicate that they have always paid attention to this field of research. The keywords “polybrominated diphenyl ethers,” “heavy metals,” “exposure,” and “management” are frequently used in the study. Meanwhile, over time, the focus of the research field shifted to the health and circular economy implications of heavy metal exposure and accumulation due to e-waste. It indicates that the research hotspot should include e-waste, heavy metal exposure, and the corresponding management. Future research directions may include the exposure probability of occupational and non-occupational groups, the exposure probability and degree of different occupational groups, the application of nanomaterials and other emerging materials to replace heavy metal materials in electronic products, and the development of environmentally friendly and effective recycling methods for electronic pollutants (56). There has been an increase in the attention paid to biohydrometallurgy because the main advantages of biohydrometallurgy compared to other methods include low operating and maintenance costs, a low energy input, the use of natural resources such as air and water, a low environmental impact, and operation at ambient temperature and pressure, which is more in line with the principles of the circular economy (57). The study by Khalid et al. (37) revealed that several microorganisms can bio-transform or mineralize PCBs under aerobic or anaerobic conditions.

However, this analysis has some drawbacks. First, only the WoS database was used for the search. It would have been preferable to incorporate these findings with those from additional databases; WoS was the most widely used database in scientometrics, and the majority of bibliometric tools can recognize the formats of files exported from WoS. Second, we restricted the inclusion criteria to English-language studies of WoS. Then, to better display the keywords, the keywords that appeared more than five times in the network were considered. Due to the occurrence of fewer than five appearances of relevant keywords that first occurred in recent years, such as volatile organic compounds and biomarkers, we might have missed the most recent study trends.

## Conclusion

This study explored the progress of research on the relationship between e-waste and health by using a quantitative scientometric analysis. We believe that the results of this study will help professionals identify patterns and trends, although it may not accurately represent the micro aspects of these results. Based on the results from the scientometric analysis, the center country of the research field was China. The most active journal was “Science of the Total Environment,” and the most active authors were Huo X and Xu XJ. According to citations, co-citations, and keyword analysis, the current research focus might shift to the health and circular economy implications of exposure to heavy metals, polychlorinated biphenyls (PCBs), polybrominated biphenyl ethers (PBDEs), and poly- and perfluorinated alkyl substances (PFAS), and relevant managements, such as exploring new environmentally friendly and recycling methods of e-waste and developing relatively mature approaches, such as hydrometallurgy and more. This paper identified trends in electronics and health and provided researchers with some highly cited references and potential research directions that are instructive for the field of public health. This study can also help researchers and decision-makers in the e-waste research field better understand the research directions and make scientific choices.

## Equations


$$\begin{array}{l} \text{The growth rate between year x and year y}\\ =\left(\text{nx }-\text{ ny}\right)÷\text{nx}\times 100\% \end{array}$$

n, the number of publications in year x or y.

## Data availability statement

The raw data supporting the conclusions of this article will be made available by the authors, without undue reservation.

## Author contributions

HT: idea conception and design and original draft writing. JW, DZ, and QY: data collection and methodology. HT and LC: data processing and visualization by software. LC, ZJ, and JC: supervision and amendment. YC and ZL: funding acquisition, resource allocation, and project administration. All authors contributed to the article and approved the submitted version.
