# Supplementary material for: Global research into the relationship between electronic waste and health over the last 10 years: A scientometric analysis
Source: Front Public Health. 2023 Jan 4;10:1069172. doi: 10.3389/fpubh.2022.1069172 (PMC9846604; doi:10.3389/fpubh.2022.1069172)
Supplement: Supplementary file 1 [file Data_Sheet_1.docx]

| **Sources** | **Articles** |
| --- | --- |
| Science of the Total Environment | 56 |
| Environmental Science and Pollution Research | 44 |
| Environment International | 32 |
| Chemosphere | 27 |
| Environmental Pollution | 26 |
| International Journal of Environmental Research and Public Health | 24 |
| Waste Management | 20 |
| Journal of Hazardous Materials | 18 |
| Journal of Cleaner Production | 17 |
| Resources Conservation and Recycling | 16 |

**Supplementary Table 1. Top 10 most relevant source**


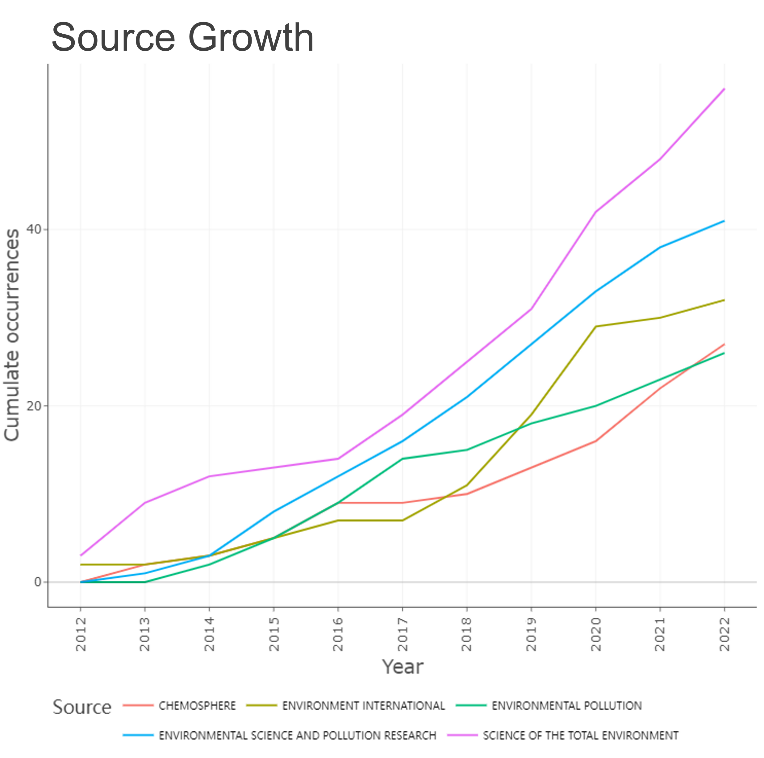


**Supplementary Figure 1. Source growth dynamic**


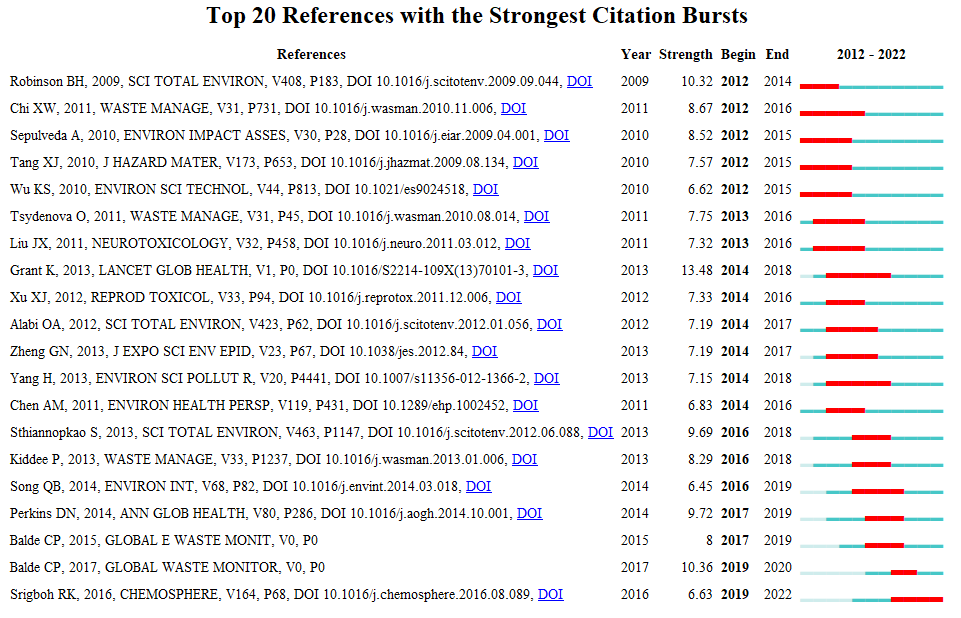


**Supplementary Figure 2. Top 20 References with the strongest citation bursts**


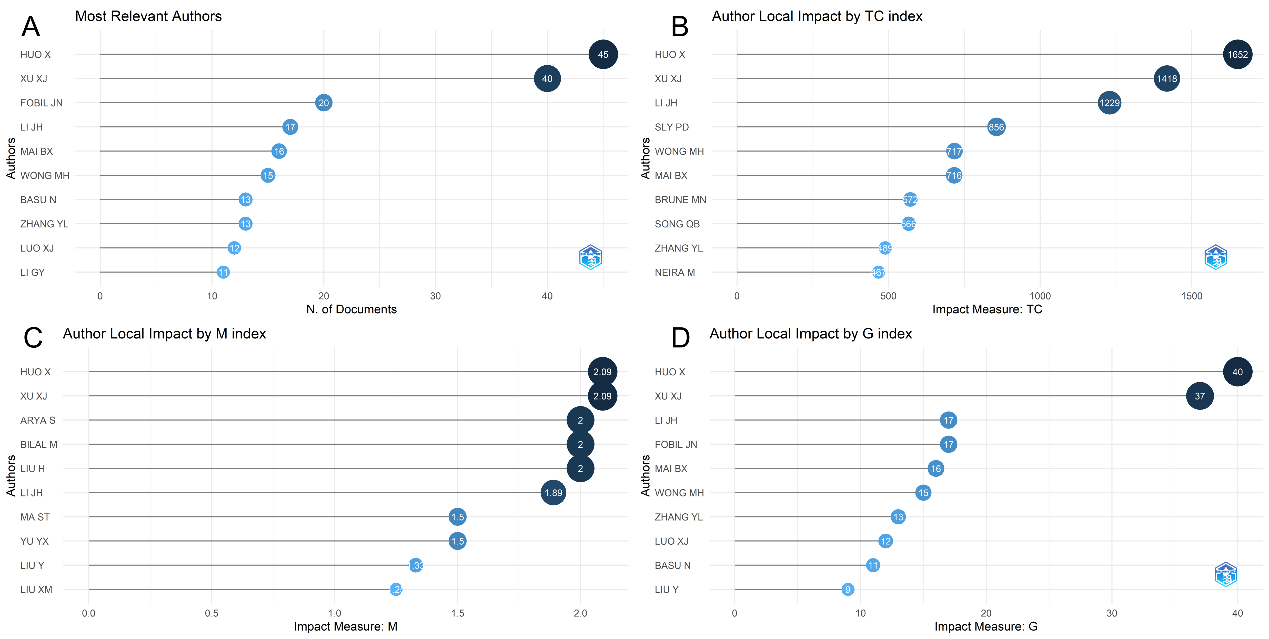


**Supplementary Figure 3. (A) Top 10 authors ranked by number of documents (B) Top 10 authors ranked by total citation (C) Top 10 authors ranked by m-index (D) Top 10 authors ranked by g-index**
